# CD39^+^ Regulatory T Cells Attenuate Lipopolysaccharide-Induced Acute Lung Injury *via* Autophagy and the ERK/FOS Pathway

**DOI:** 10.3389/fimmu.2020.602605

**Published:** 2021-01-08

**Authors:** Cen Chen, Xinying Li, Chuling Li, Jiajia Jin, Donghui Wang, Yuan Zhao, Yanli Gu, Meizi Chen, Suhua Zhu, Hongbing Liu, Tangfeng Lv, Fang Zhang, Yong Song

**Affiliations:** ^1^ Department of Respiratory and Critical Care Medicine, Jinling Hospital, The First School of Clinical Medicine, Southern Medical University, Nanjing, China; ^2^ Department of Respiratory and Critical Care Medicine, Jinling Hospital, Medical School of Nanjing University, Nanjing, China; ^3^ Department of Respiratory and Critical Care Medicine, Jinling Hospital, Nanjing Medical University, Nanjing, China; ^4^ Jiangsu Provincial Key Laboratory of Critical Care Medicine, Department of Critical Care Medicine, Zhongda Hospital, School of Medicine, Southeast University, Nanjing, China; ^5^ Department of General Internal Medicine, The First People’s Hospital of Chenzhou, Chenzhou, China

**Keywords:** acute respiratory distress syndrome, CD39, regulatory T cells, acute lung injury, adoptive transfer, autography, ERK, FOS

## Abstract

Acute respiratory distress syndrome (ARDS) is characterized by an uncontrollable cytokine storm, which is associated with high mortality due to lack of effective treatment. Regulatory T cells (Tregs) play an indispensable role in maintaining immune homeostasis and CD39 is considered as a functional cell marker of Tregs. In this study, we aimed to evaluate the effect of CD39^+^ Tregs on acute lung injury (ALI) and investigate the frequency of CD39^+^ Tregs in ARDS patients. We found that after lipopolysaccharide (LPS) treatment, CD39^−/−^ mice exhibited more severe inflammation and wild type (WT) mice exhibited a decreased frequency of CD39^+^ Tregs in the peripheral blood. Furthermore, CD39^+^ Tregs had a protective effect on LPS-induced inflammation *in vitro* and the adoptive transfer of CD39^+^ Tregs had a therapeutic effect on ALI *in vivo*. We further sought to explore the mechanisms that affect CD39 expression on Tregs. LPS-induced inflammation in the lung impaired the immunosuppressive effect of Tregs *via* the autophagy-mediated downregulation of CD39. In addition, CD39 induced the expression of itself in Tregs *via* activating the ERK1/2-FOS pathway. Consistent with this finding, the frequency of CD39^+^ Tregs was also decreased in the peripheral blood of ARDS patients and was positively correlated with disease severity. Our results suggested that the adoptive transfer of CD39^+^ Tregs may provide a novel method for the clinical prevention and treatment of ARDS.

## Introduction

Acute respiratory distress syndrome (ARDS), previously known as acute lung injury (ALI), is a type of acute diffuse, inflammatory injury of the lung that leads to increased pulmonary vascular permeability and lung weight, as well as the loss of aerated lung tissue (1). Since the conception of ARDS was proposed in 1967 (2), there are a limited number of effective treatments aimed at pathogenesis. Despite considerable progress in the understanding of molecular mechanisms, advances in ventilatory strategies, and general care of critically ill patients, the mortality remains unacceptably high at 35–45% (3). Currently, supportive care with low tidal volume ventilation is still the predominant therapeutic strategy for ARDS (4). Several novel pharmacological therapeutics, including β-2 agonists (5), statins (6, 7), aspirin (8), nitric oxide (9), and keratinocyte growth factor (10) also showed no obvious clinical benefit. To date, while cell-based approaches, primarily using mesenchymal stem/stromal cells represents the most promising therapy, research remains stagnated in early-phase clinical trials (11). Therefore, there is an urgency to identify innovative and effective therapeutics that can be used to target ARDS.

Inflammatory and hypoxic conditions in ARDS lead to the increased release of adenosine triphosphate (ATP)/adenosine diphosphate (ADP). Extracellular ATP (eATP) acts as danger-associated molecular patterns (DAMPs), binds to purinergic receptors, thereby triggering signaling cascades to induce an inflammatory response (12). ATP/ADP is then metabolized to adenosine monophosphate (AMP) *via* CD39 (ectonucleoside triphosphate diphosphohydrolase-1, ENTPD1). AMP is further hydrolyzed to adenosine *via* CD73 (ecto-5’-nucleotidase). Adenosine is a endogenous molecule that inhibits T cell responses by activating G-protein-coupled P1 receptors expressed on immune cells (e.g., macrophages, dendritic cells, and lymphocytes) (13). In an acute setting, adenosine signaling serves an anti-inflammatory, tissue-protective role (14). CD39, not CD73, is the rate-limiting enzyme in the ATP/ADP-AMP-adenosine pathway. Taken together, CD39 plays a non-ignorable part in the shift from an ATP-mediated pro-inflammatory milieu to an immunosuppressive setting driven by adenosine (15). CD39 has been reported to be expressed on the surface of human and murine Tregs (16, 17). Previous studies have revealed a close relationship between CD39 and Tregs (18–20) and CD39 is one of the most pronounced overlapping genes related to the suppressive function of Tregs (21). The ATP- CD39- CD73- adenosine axis contributes to Foxp3^+^ CD4^+^ suppressor T cell activity (22). CD39^+^ Tregs present stronger stability and function under inflammatory conditions (23). The role of CD39 on Tregs in limiting tissue injury has been studied in myocardial infarction (24) and benign prostate hyperplasia (25). All of these findings suggest a potential role of CD39^+^ Tregs in acute lung inflammatory disease.

In the present study, to address the functional role of CD39^+^ Tregs during the ARDS, we used CD39 deficient mice. Subsequently, we evaluated the adoptive transfer CD39^+^ Tregs for use in the immunotherapy of ALI mice and explored the potential mechanism affecting CD39 expression on Tregs. We investigated the effect of CD39^+^ Tregs in ARDS patients. Our findings implied that CD39^+^ Tregs played a protective role in ARDS and may represent a potential therapeutic target.

## Materials and Methods

### Experimental Animals

CD39^−/−^ and C57BL/6J mice (GemPharmatech, CHN) were housed with free access to sterile water and food under 12-h light:12-h dark cycle conditions. The animal protocols were performed in accordance with the China Council on Animal Care and approved by the Institutional Animal Care and Use Committee of Jingling Hospital.

### ALI Model and Adoptive Transfer

To establish the ALI model, male mice, aged 8 to 10 weeks, were anesthetized with pentobarbital (3 mg/kg) before the procedure. LPS (Sigma-Aldrich, USA) at 10 mg/kg (for an evaluation of lung injury and inflammation) or 20 mg/kg (for an analysis of survival rate) were intratracheally instilled. Mice in the control group were intratracheally instilled with PBS. To investigate the effect of CD39^+^ Tregs for ALI, different isolated Tregs subsets (5 × 10^5^) were injected by tail vein 30 min before the instillation of LPS.

### Lung Histology and Lung Injury Scoring

Mice were killed by a pentobarbital overdose followed by exsanguination. The lungs were fixed in a 4% paraformaldehyde solution overnight and subsequently embedded in paraffin, sectioned to 5 μm-thick sections, and stained with hematoxylin and eosin (H&E) as described previously (26) for histopathological scoring or anti-myeloperoxidase (MPO) antibodies for immunohistochemistry (IHC) analysis.

Two random H&E-stained tissue sections were examined by a pathologist who was blinded to the genetic background and treatment of the mice. ALI was scored as described previously (27), in accordance with the following criteria: 1) alveolar congestion; 2) hemorrhage; 3) infiltration or aggregation of neutrophils in the airspace or vessel wall; and 4) thickness of the alveolar wall/hyaline membrane formation. For each subject, a five-point scale was applied: 0, minimal (little) damage; 1+, mild damage; 2+, moderate damage; 3+, severe damage; and 4+, maximal damage. Points were totaled and expressed as the median ± range.

### Bronchoalveolar Lavage Fluid (BALF) and Blood Analysis

BALF was obtained as previously described and 1 ml PBS was intratracheally instilled into the lung and then lavaged three times. All of the removed fluid was immediately centrifuged (300 × g, 5 min). The total cells were stained using the Papanicolaou method to obtain cell counts. The supernatant was collected for cytokine analysis. After sacrificing the mice, the blood from the vena cava was immediately collected (300 × g for 5 min). The cells were prepared for further flow cytometry analysis and the plasma was used for cytokine analysis.

### Flow Cytometry

Cells from the BALF were stained with surface markers, including PE-Cy7 conjugated anti-CD45 (BD Biosciences, CA, USA), AF488 conjugated anti-CD11b (BD Biosciences, CA, USA), and PE conjugated anti-Ly6G (BD Biosciences, CA, USA). Cells from blood and spleen were stained with various surface markers, including PE-Cy7 conjugated anti-CD45 (BD Biosciences, CA, USA), Percp-cy5.5 conjugated anti-CD3 (BD Biosciences, CA, USA), FITC conjugated anti-CD4 (BD Biosciences, CA, USA), PE conjugated anti-CD25 (BD Biosciences, CA, USA), and APC conjugated anti-CD39 (BD Biosciences, CA, USA).

Peripheral blood mononuclear cells (PBMCs) from ARDS patients and healthy donors were stained with PerCP conjugated anti-CD3 (BD Biosciences, CA, USA), FITC conjugated anti-CD4 (BD Biosciences, CA, USA), APC conjugated anti-Foxp3 (BD Biosciences, CA, USA), and PE conjugated anti-CD39 (BD Biosciences, CA, USA). This study was approved by the Jinling Hospital Ethics Review Committee and written informed consent was provided by all subjects or their legal representatives.

### Cytokine Analysis

Concentrations of IL-6, IL-1β, and TNF-α in the BALF, plasma, and supernatant of the co-cultured system were measured using an ELISA kit (eBioscience, USA) according to the manufacturer’s recommendations.

### Immunofluorescence

Immunofluorescent (IF) staining was used to show the changes in CD39^+^ Tregs in the mouse lung tissue. Tissue sections were stained with primary antibodies against Foxp3 (Abcam, USA) and CD39 (Abcam, USA). The sections were then stained with an AF594 donkey anti-mouse IgG antibody (Abcam, USA), AF488 donkey anti-rabbit IgG antibody (Abcam, USA), and 4’6-diamidino-2-phenylindole (DAPI, Abcam, USA).

### Tregs Isolation and Stimulation

Tregs from mouse spleens and blood were isolated by a CD4^+^CD25^+^ Regulatory T-cell Isolation Kit (Miltenyi Biotec, GEM) according to the manufacturer’s recommendations. The Tregs were stained with APC conjugated anti-CD39 (BD Biosciences, CA, USA) and subjected to flow cytometry.

Tregs isolated from WT and CD39^−/−^ mice were stimulated *in vitro* for 3 days with 4 ng/ml recombinant mouse IL-2 (Absin, CHN) in 24-well plates, which were precoated with 1 μg/ml anti-mouse CD3 (Absin, CHN) and anti-mouse CD28 Abs (Absin, CHN). The purity of the isolated Tregs was >95% by a flow cytometric analysis with FITC conjugated anti-CD4 (BD Biosciences, CA, USA) and PE conjugated anti-CD25 (Miltenyi Biotec, GEM).

### *In Vitro* Suppression Assay

Different activated Tregs subsets were cultured with RAW264.7 macrophages in DMEM medium (Bioind, ISR) containing 10% fetal bovine serum (Bioind, ISR) 30 min before LPS stimulation (1 μg/ml). Supernatants were collected at the indicated time points (300 × g for 5 min), flash-frozen, and stored at −80°C for further analysis. The cells that had adhered to the tube after centrifugation and the cells adhered to the plate were used for transcriptional analysis.

### Quantitative Real-Time Reverse Transcription Polymerase Chain Reaction (qRT-PCR)

The level of IL-6, IL-1β, TNF-α, and CD39 mRNA expression were detected by qRT-PCR. Total RNA was isolated from RAW264.7 or Tregs using TRIzol Reagent (TaKaRa, Japan). cDNA synthesis was performed using a PrimeScriptTM RT reagent Kit (Takara, Japan). qRT-PCR was then performed using ABIQ3 (Applied Biosystems, USA) and a SYBER Prime ScriptTM RT Reagent Kit (Takara, Japan) according to the manufacturer’s recommendations. The following primer sequences were used: murine IL-6 5’-TAGTCCTTCCTACCCCAATTTCC-3’ and 5’-TTGGTCCTTAGCCACTCCTTC-3’ (sense/antisense); IL-1β 5’-GCAACTGTTCCTGAACTCAACT-3’ and 5’-ATCTTTTGGGGTCCGTCAACT-3’ (sense/antisense); TNF-α 5ʹ- CCCTCACACTCAGATCATCTTCT-3’ and 5’- GCTACGACGTGGGCTACAG-3’ (sense/antisense); CD39 5’-TACCACCCCATCTGGTCATT-3’ and 5’-GGACGTTTTGTT TGGTTGGT-3’ (sense/antisense). Glyceraldehyde-3-phosphate dehydrogenase (GAPDH) and β-actin were used as the reference genes. The primer sequences for murine GAPDH and β-actin were 5’-AGGTCATCCCAGAGCTGAACG-3’ and 5’-CACCCTGTTGCTGTAGCCGTAT-3’ (sense/antisense) and 5’-ACAT TGGCATGGCTTTGTTT-3’ and 5’-GTTTGCTCCAACCAACTGCT-3’ (sense/antisense), respectively. The levels of mRNA expression were calculated using the 2-ΔΔCT method.

### Western Blotting

In the Western blot analysis, proteins were extracted from Tregs using a protein extraction kit (KeyGene, NED). The extracted proteins were added to 10% sodium dodecyl sulfate-polyacrylamide gel electrophoresis followed by migration with 120 V electric tension, and transferred onto nitrocellulose membranes. The membranes were blocked with 5% skimmed milk powder in Tris-buffered saline containing Tween 20 (TBST), and incubated with specific primary antibodies (Abcam, USA) at 4°C overnight. The following day, the membranes were washed for 15 min three times in TBST, and incubated with secondary antibodies (Abcam, USA) for 2 h at room temperature. HRP Substrate (Millipore, USA) was used to visualize the protein bands, and the band intensities were quantified using Image Pro plus software (Mediacy, USA). For more detailed steps, please refer to the antibody specification and protocol description.

### GFP-mRFP-LC3 Detection by Confocal Microscopy

Lentivirus particles carrying GFP-mRFP-LC3 (double-labeled fusion gene with LC3) were generated by Shanghai Genechem Company (Shanghai, China) and used to infect Tregs according to the manufacturer’s instructions. Tregs were evaluated by confocal microscopy (ZEISS, GEM) after Hoechst 33342 staining. The number of puncta per cell was calculated in six high-power field areas (63 × oil-immersion objectives).

### Chromatin Immunoprecipitation (ChIP) Assay

A ChIP assay was carried out using an EZChip kit (Millipore, USA) according to the manufacturer’s protocol. Briefly, samples from the experimental and input groups were cross-linked with 1% formaldehyde for 10 min at room temperature, and glycine was added to terminate the reaction. A DNA-protein complex was formed by this cross-linking. An FOS antibody (Abcam, USA) was added to the samples from the experimental group to fully form the target DNA fragment/FOS/FOS antibody complex. The complex was precipitated with protein A/G magnetic beads and washed three times to remove any non-specific DNA, the elution buffer was added to elute the DNA. The cross-linking buffer was decoupled overnight, the DNA in the product was extracted, and purified by DNA extraction kit (Qiagen, USA). The target DNA was detected by PCR, and the target DNA results was presented as the % of input. The primers were designed based on the 50 bp around the predictive site on the CD39 promoter for PCR detection.

### Luciferase Reporter Assay

The CD39 promoter regions (region 1: −700 to −600 bp; region 2: −1,500 to −1,400 bp) were cloned into pGL4 luciferase reporter plasmids (ViGene Biosciences, CHN). Control plasmids were constructed with a pRL-TK vector containing Renilla luciferase. FOS-overexpressed plasmids were constructed using the pCDNA3.1 vector (ViGene Biosciences, CHN). Next, 293T cells were plated into 24-well plates and cultured until the cell density reached 60%. The promoter (300 ng) and control plasmids (30 ng) were co-transfected with or without FOS-overexpressed plasmids (600 ng) into the cells using Lipofectamine 3000 reagent (Thermo Fisher Scientific, USA). After culturing for 48 h, the relative luciferase activity was determined using the dual-luciferase reporter assay kit in accordance with the manufacturer’s instructions (Promega, USA). The level of fluorescence was detected using a microplate reader.

### Statistical Analysis

Data are expressed as the mean ± SD or percentage in the figures. Survival analysis was performed using the Kaplan-Meier method. Continuous data were tested using an unpaired Student’s t-test between two groups and a one-way ANOVA was performed for multiple comparisons. A Mann-Whitney U test and a Kruskal-Walls test was performed for the nonparametric data. All analyses were performed using SPSS 26.0 (IBM, USA). P < 0.05 was considered to be significant.

## Results

### A CD39 Deficiency Exacerbates LPS-Induced ALI in Mice

**Figure 1:**
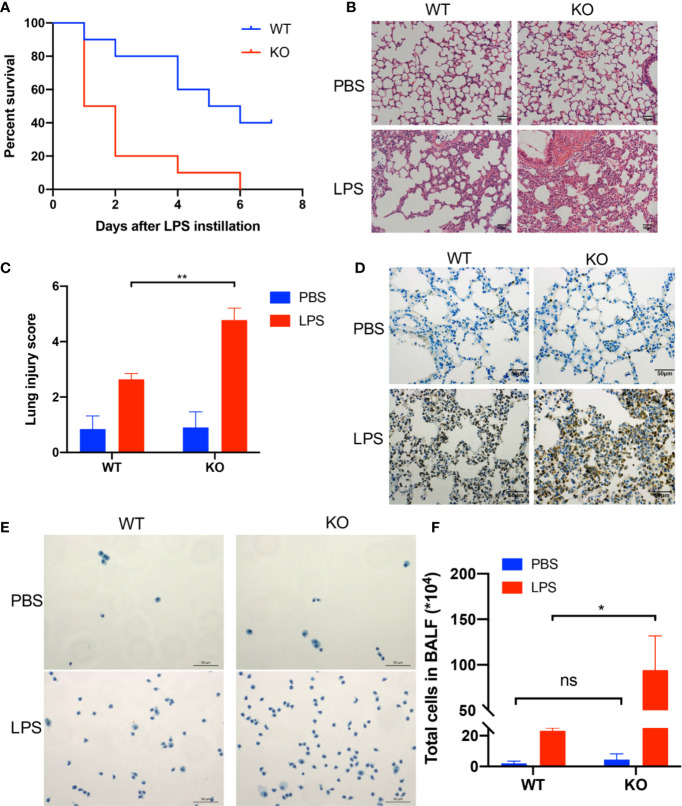
A CD39 deficiency exacerbated LPS-induced acute lung injury in mice. **(A)** Survival curve of WT mice and CD39^−/−^ mice following lung injury. Log rank test, P = 0.005. **(B)** and **(C)** H&E staining of the lung tissue (200×) and lung injury score of each group (n = 5). **(D)** Representative MPO immunohistochemistry images of each group. **(E)** Representative images of the total cells in the BALF (400×). **(F)** The total BALF cells in each group were counted (n = 3). *P < 0.05.

**Figure 2:**
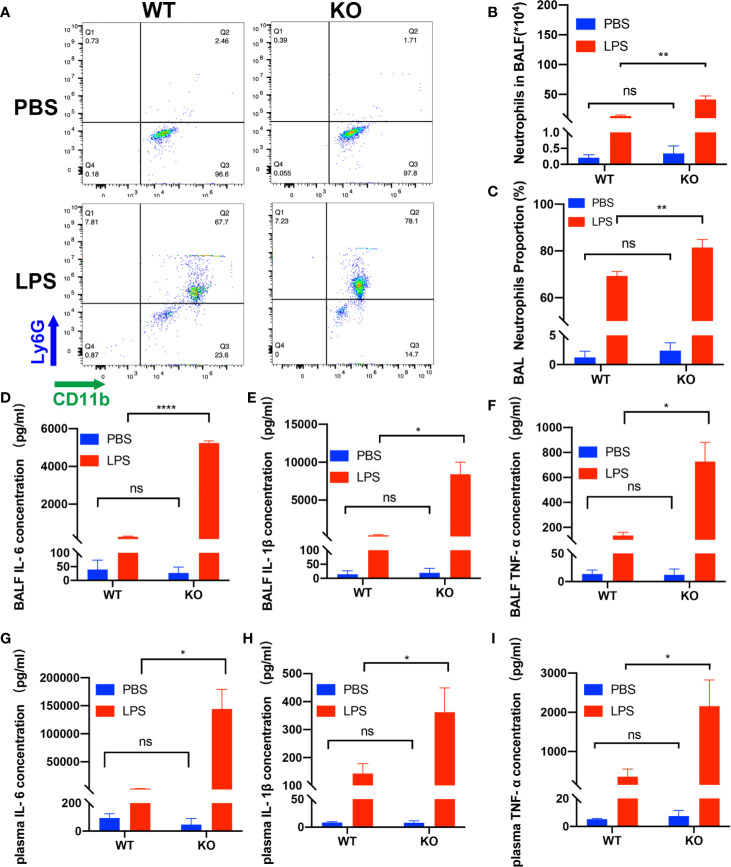
Deficiency of CD39 increases inflammation in ALI mice. **(A)** Representative FACS analysis shows neutrophils in the BALF. The total BALF cells were first gated on CD45-positive cells, and then gated on Ly6G- and CD11b-positive cells. **(B, C)** Analysis of neutrophils in the BALF of each group (n = 3). **(D–F)** The BALF concentration of IL-6, IL-1β, and TNF-α in each group (n = 3). **(G–I)** The plasma concentration of IL-6, IL-1β, and TNF-α in each group (n = 3). *P < 0.05; ****P < 0.0001.

First, we examined the role of CD39 on ALI *in vivo*. For this purpose, we used previously characterized CD39^−/−^ mice (28) or age-, sex-, and weight-matched littermate controls and induced ALI *via* an intratracheal administration of LPS. To investigate the effect of CD39 on the survival of ALI mice, we challenged mice with 20 mg/kg LPS. As shown in **Figure 1A**, CD39^−/−^ mice started to die on Day 1, and none survived to Day 7. The mortality of the WT group was 60%. A CD39 deficiency significantly impaired the survival of ALI mice. To evaluate the level of lung injury and inflammation, mice were subjected to an intratracheal injection of 10 mg/kg LPS or PBS as a control. After 12 h, the lung tissue was subjected to H&E staining and scored to determine the severity of tissue injury. **Figure 1B** shows the typical manifestations of murine ALI, including marked infiltration or aggregation of neutrophils, thickening of the alveolar wall, and hemorrhaging. Regarding the lung injury score (**Figure 1C**), a CD39 deficiency significantly enhanced the degree of lung injury compared with that of the WT mice. The IHC of MPO further proved that additional infiltration of polymorphonuclear neutrophils (PMN) occurred in CD39^−/−^ mice (**Figure 1D**). Moreover, the total number of BALF cells (**Figures 1E, F**) and the subset of neutrophils (**Figures 2A–C**) were both significantly increased in CD39^−/−^ mice. Furthermore, a significant increase of BALF and plasma inflammatory cytokine levels was observed in CD39^−/−^ mice, including IL-6, IL-1β, and TNF-α (**Figures 2D–I**). Taken together, these data revealed that CD39 played a functional role in ALI resolution.

### CD39 Expression on Tregs Is Decreased in ALI Mice

**Figure 3:**
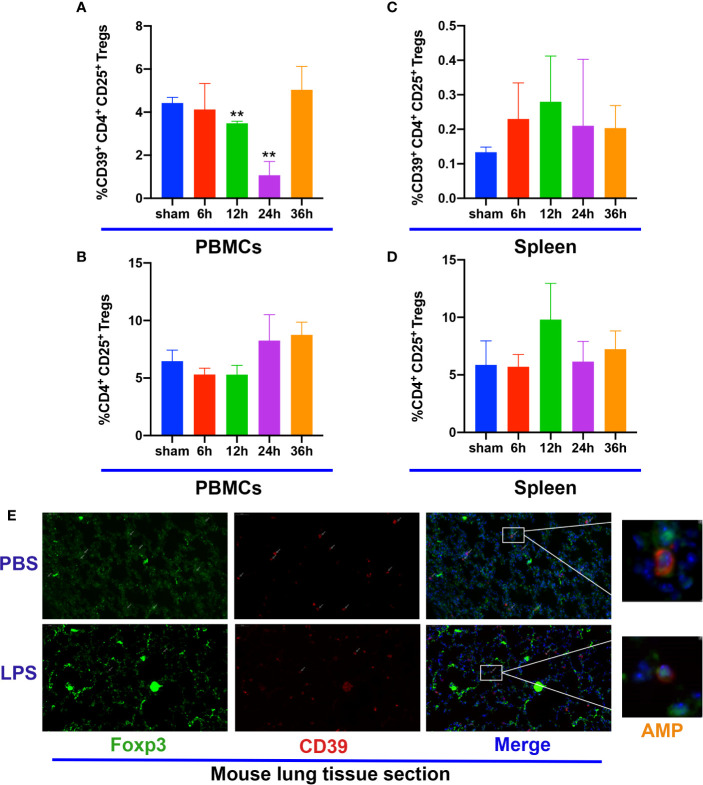
CD39 expression on Tregs is decreased in ALI mice. **(A, C)** Analysis of CD39^+^ Tregs in PBMCs and the spleen at different time points. **(B, D)** Analysis of CD4^+^CD25^+^ Tregs in PBMCs and the spleen at different time points. **(E)** Representative immunofluorescence images of Foxp3 and CD39 expression in the lung tissue of ALI mice at 12 h. ^*^P < 0.05; ^***^P < 0.001.

Previous studies have shown that Tregs play a key role in the resolution ALI (29), whereas other studies demonstrate that Treg-dependent immune functions are linked to CD39 expression (15). To investigate the kinetics of Tregs after stimulation with LPS in WT mice, the population of Tregs in the blood and spleen were analyzed by flow cytometry. Foxp3 and CD39 expression in lung tissues were examined by immunofluorescence. The percentage of CD39 expression on CD4^+^CD25^+^ Tregs among CD4^+^ T-cells within the blood had a tendency to decrease at 12 h following LPS stimulation, continued to become down-regulated for 24 h and spontaneous recovery for 36 h (**Figure 3A**). In contrast, the percentage of CD4^+^CD25^+^ Tregs among CD4^+^ T cells did not exhibit a significant difference (**Figure 3B**); however, both the percentage of CD39 expression on CD4^+^CD25^+^ Tregs and the percentage of CD4^+^CD25^+^ Tregs among CD4^+^ T cells within the spleen were similar between the different groups (**Figures 3C, D**). Moreover, CD39^+^ Tregs in the lung tissue at 12 h also showed a decrease (**Figure 3E**). Therefore, our data indicate a potential role for CD39^+^ Tregs in the course of ALI.

### CD39^+^ Tregs Decreased LPS-Induced Inflammation *In Vitro*

**Figure 4:**
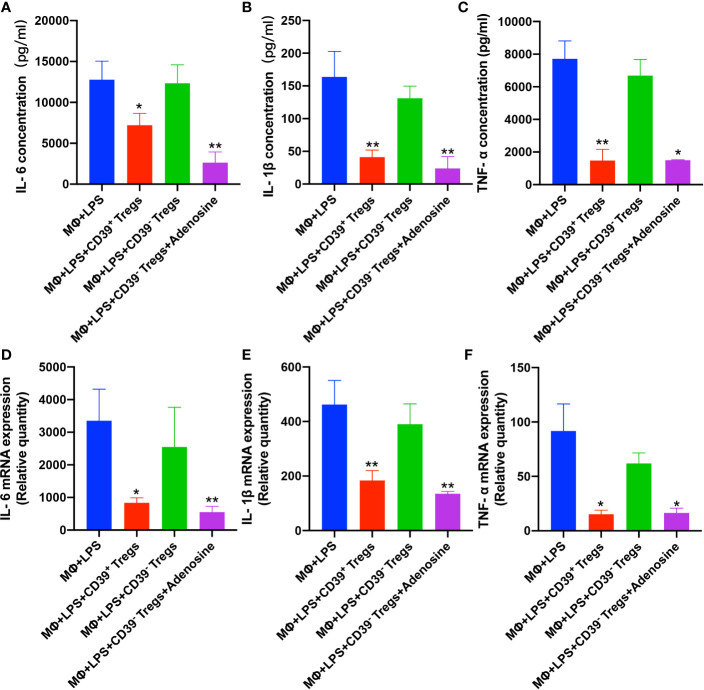
CD39^+^ Tregs decreased LPS-induced inflammation *in vitro*. Both CD39^+^ and CD39^-^ Tregs were co-cultured with RAW264.7 cells in the absence of LPS after activation. The level of LPS-induced protein expression of **(A)** IL-6, **(B)** IL-1β, and **(C)** TNF-α in the supernatant were determined by an ELISA. The level of LPS-induced **(D)** IL-6, **(E)** IL-1β, and **(F)** TNF-α mRNA expression in macrophages were analyzed by qRT-PCR. ^*^P < 0.05; ^**^P < 0.01.

To further substantiate the role of CD39 expressed on Tregs during inflammation, we performed a set of *in vitro* experiments. First, we harvested CD4^+^CD25^+^ T cells from the spleen and characterized the CD39^+^ (from WT mice) and CD39^−^ (from CD39^−/−^ mice) Treg populations. The Tregs were activated with IL-2, anti-CD3, and anti-CD28 for 3 days. The activated Tregs were then incubated with RAW264.7 cells in the presence or absence of LPS. Following LPS stimulation, both the level of mRNA and protein expression of IL-6, IL-1β, and TNF-α were increased in the CD39^-^ Treg group (**Figures 4A–F**). Moreover, a preincubation with adenosine could reduce these proinflammatory cytokines. These results suggested that the upregulation of CD39 on Tregs exhibited anti-inflammatory activity.

### Adoptive Transfer of CD39^+^ Tregs Protects Against LPS-Induced ALI

**Figure 5:**
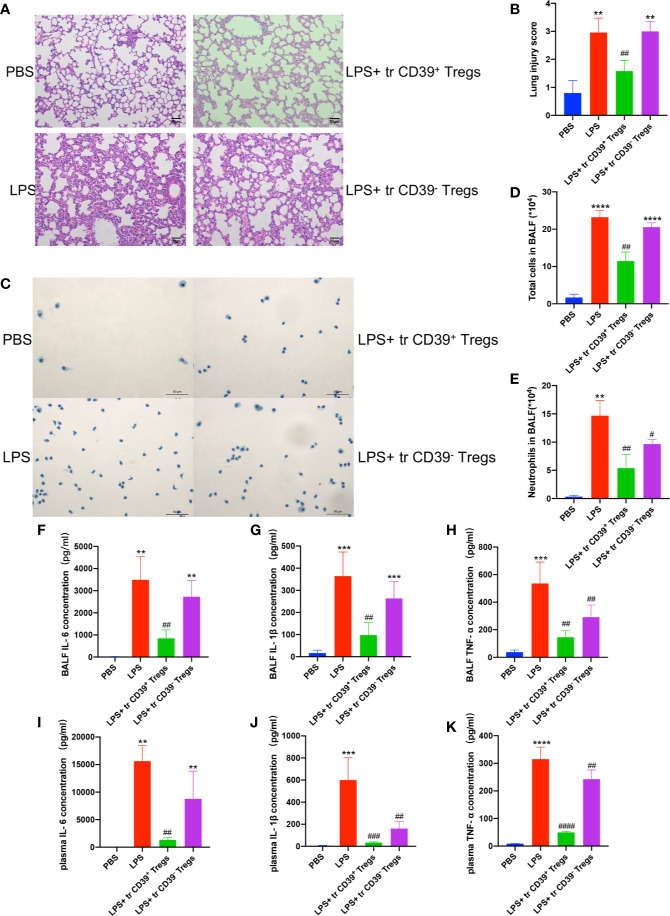
Adoptive transfer of CD39^+^ Tregs protects against LPS-induced ALI. **(A, B)** H&E staining of the lung tissue (200×) and lung injury score of each group (n = 5). **(C)** Representative images of the total cells in the BALF (400×). **(D)** Total cells in the BALF were counted in each group (n = 3). **(E)** Analysis of neutrophils in the BALF of each group (n = 3). The concentration of IL-6 **(F, I)** IL-1β **(G, J)**, and TNF-α **(H, K)** in the BALF and plasma in each group (n = 3). ^**^P < 0.01; ^***^P < 0.001; ^****^P < 0.0001; ^##^P < 0.01; ^###^P < 0.001; ^####^P < 0.0001 *vs.* LPS.

To address the functional role of Treg-dependent CD39 during ALI, we next performed adoptive transfer studies. A total of 5 × 10^5^ CD4^+^CD25^+^ T cells were separated from the spleen with a purity above 95% (**Supplementary Figure 2**), and transferred into recipient mice prior to intratracheal LPS stimulation. As expected, mice treated with CD39^+^ Tregs exhibited attenuated ALI. Lung histology and lung injury scoring reflected a progressive resolution in mice receiving CD39^+^ Tregs compared with CD39^−^ Tregs (**Figures 5A, B**). The lavage from mice receiving CD39^−^ Tregs contained significantly higher number of total cells (**Figures 5C, D**) and neutrophils (**Figure 5E**). In line with the findings of the *in vitro* studies, the level of IL-6, IL-1β, and TNF-α expression was decreased in the BALF and plasma in those mice injected with CD39^+^ Tregs (**Figures 5F–K**). Taken together, these studies indicated the therapeutic potential of CD39^+^ Tregs for ALI.

### CD39 Self-Upregulates Its Expression *via* Activating the ERK/FOS Pathway in Tregs

**Figure 6:**
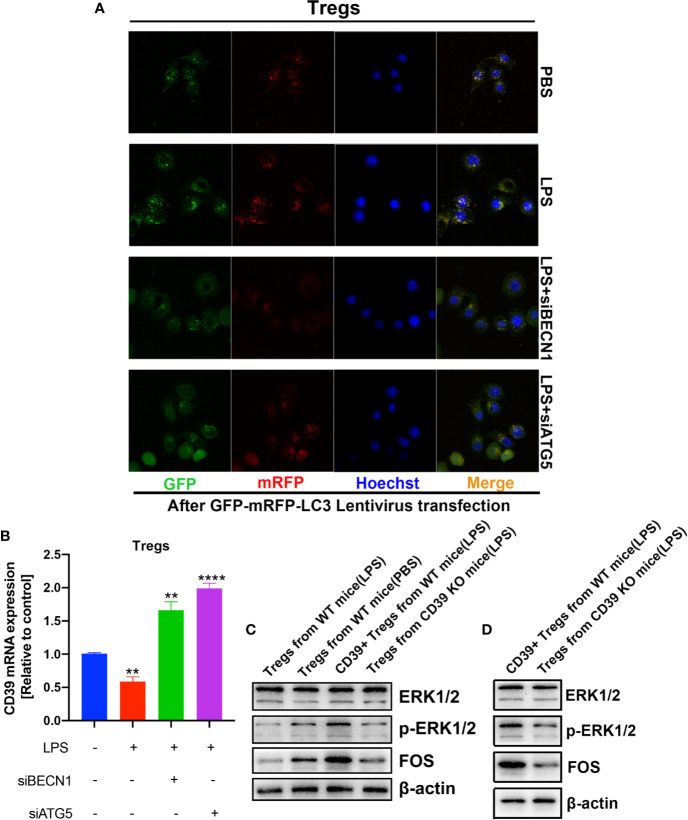
Autophagy and the ERK/FOS pathway co-mediated CD39 expression on Tregs during inflammation. After isolation and activation *in vitro*, Tregs transfected with the GFP-mRFP-LC3 lentivirus were treated with LPS or PBS in a co-culture system. **(A)** Confocal microscopy was performed to determine the level of LC3 expression. **(B)** CD39 expression was measured by RT-PCR. **(C, D)** Western blotting to determine the level of phosphorylated ERK1/2 and FOS in Tregs under different conditions. ^**^P < 0.01; ^****^P < 0.0001.

**Figure 7:**
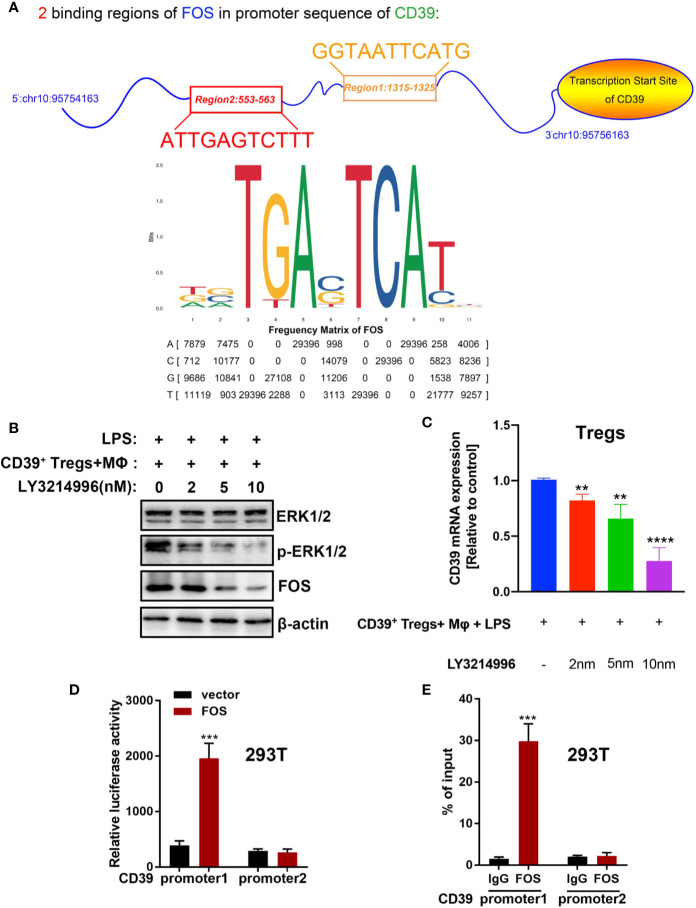
CD39 self-upregulates its expression *via* activating the ERK/FOS pathway in Tregs. **(A)** A bioinformatics analysis showed that two potential binding sites of FOS are located in the CD39 promoter. **(B)** Western blotting for the phosphorylation of ERK1/2 and FOS and **(C)** the expression of CD39 in Tregs following treatment with different concentrations of LY3214996. **(D)** A promoter luciferase assay was carried out after co-transfecting 293T cells with CD39 promoter reporter plasmids containing a different FOS binding region and FOS-overexpressing plasmids. Region 1: −700 bp to −600 bp of the CD39 promoter; region 2: −1,500 bp to −1,400 bp of the CD39 promoter. **(E)** ChIP-PCR was used to validate the binding site of FOS on the CD39 promoter in 293T cells using specific primers. Region 1: −685 bp to −675 bp of the CD39 promoter; region 2: −1,447 bp to −1,437 bp of the CD39 promoter. ^**^P < 0.01; ^***^P < 0.001; ^****^P < 0.0001.

Previous studies have found that cellular autophagy inhibits CD39 expression in Tregs during inflammation (30). To evaluate the effect of autophagy on CD39 in Tregs, we isolated and activated Tregs *in vitro*, then transfected the Tregs with a GFP-mRFP-LC3 lentivirus. We then determined the dual-fluorescence of LC3 following treatment with LPS or PBS in a co-culture system. As expected, LPS treatment increased the number of autophagosomes in Tregs compared to that of the PBS group. However, this promotive effect was abrogated by either transfecting the cells with small interfering RNA against either BECN1 or ATG5, two vital genes in the autophagy pathway (**Figures 6A** and **Supplementary Figure 3**). Furthermore, CD39 expression was significantly downregulated following LPS stimulation; however, this suppressive effect on CD39 was also reverted by transfection with siBECN1 or siATG5 (**Figure 6B**). This evidence suggested that LPS-induced lung inflammation impaired the immunosuppressive effect of Tregs *via* autophagy-mediated downregulation of CD39. One recent study found that CD39 protects cardiac tissue against ischemic injury *via* activation of the ERK1/2 pathway in myocardial cells (24). To investigate that whether CD39 activates the ERK1/2 pathway in Tregs, we detected activation of the ERK1/2 pathway by western blotting of Tregs collected from WT mice, CD39^−/−^ mice, and CD39^+^ Tregs enriched from WT mice following *in vitro* treatment with PBS or LPS. We found that the level of phosphorylated ERK1/2 and its downstream transcription factor, FOS, were significantly increased in CD39^+^ Tregs compared to that of other Tregs (**Figure 6C**). In addition, we observed that ERK1/2 phosphorylation and FOS expression was decreased in Tregs extracted from CD39^−/−^ mice compared to that of WT mice after an intratracheal instillation of LPS (**Figure 6D**). Intriguingly, through a bioinformatics analysis, we identified two potential binding sites of FOS on the CD39 promoter (**Figure 7A** and **Supplementary Table 1**). Moreover, CD39 expression was gradually downregulated in Tregs following 2, 5, and 10 nM LY3214996 treatment, a phosphorylated ERK1/2 inhibitor (**Figures 7B, C**). Utilizing a promoter luciferase reporter assay, we found that the fluorescence intensity was improved following co-transfecting 293T cells with FOS-overexpressed plasmids and reporter plasmids containing −700 bp to −600 bp of the CD39 promoter, but not the −1,500 bp to −1,400 bp region compared with controls (**Figure 7D**). To determine the accurate binding site of FOS on the CD39 promoter, we developed a ChIP-PCR assay. As shown in **Figure 7E**, the GGTAATTCATG (−685 bp to −675 bp) region of the CD39 promoter was detected in the ChIP extracts using an FOS antibody, but not the −1,447 bp to −1,437 bp region. Taken together, our data revealed that CD39 induced the expression of itself in Tregs *via* activating the ERK1/2-FOS pathway. This formed a positive feedback loop, as well as enhanced the protective effect of Tregs in response to inflammation.

### The Frequency of CD39^+^ Tregs Was Decreased in ARDS Patients

**Figure 8:**
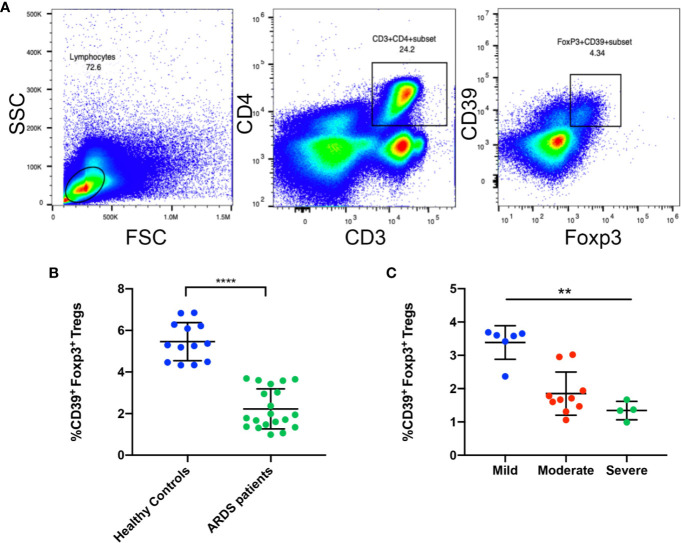
The frequency of CD39^+^ Tregs are decreased in ARDS patients. **(A)** Representative FACS analysis shows CD39^+^ Tregs in the PBMCs in ARDS patients. **(B)** Analysis of CD4^+^Foxp3^+^CD39^+^ Tregs in PBMCs from ARDS patients and healthy donors. **(C)** Analysis of CD4^+^Foxp3^+^CD39^+^ Tregs in different groups of ARDS patients. ^**^P < 0.01; ^****^P < 0.0001.

After the above investigation of CD39^+^ Tregs was conducted in an animal model, we sought to determine the regulation of CD39^+^ Tregs in ARDS patients. A total of 20 patients and 13 healthy controls were enrolled in the present study. The general characteristics of the patients and healthy controls are presented in **Table 1**. Blood samples were collected within 24 h after the patients were diagnosed with ARDS. **Figure 8A** shows the representative FACS analysis of CD39^+^ Tregs. We found that the percentage of CD39^+^ Tregs in the CD4^+^ T cell population was significantly decreased compared to that of the healthy donors (**Figure 8B**). We classified the ARDS patients as either mild, moderate, and severe according to PaO_2_/FiO_2_. A significant difference was observed between the three groups (P = 0.002) (**Figure 8C**). As the disease worsened, the proportion of CD39^+^ Tregs gradually decreased.

**Table 1:** Characteristics of ARDS patients and healthy controls.

Clinical parameters	ARDS	Healthy controls
**Age (years)**		
<65	13	10
≥65	7	3
**Sex**		
Male	11	7
Female	9	6
**ARDS severity**		
Mild	6	/
Moderate	10	/
Severe	4	/
**Cause of admission**		
Severe pneumonia	8	/
Pancreatitis	12	/

## Discussion

ARDS is an inflammatory disorder leading to acute hypoxemic respiratory failure following inflammatory injury to the lung endothelium and alveolar epithelium (31). Inflammatory cell infiltration and the release of multiple pro-inflammatory cytokines are the core, as well as the cardinal step of ARDS. A large number of compounds have been reported to restrain LPS-induced lung inflammation, including Clara secretory cell protein (32), 13-glutathionyl, 14-hydroxydocosahexaenoic acid (33), and vitamin D (34). Unfortunately, no pharmacological interventions have proven to be effective in preclinical or clinical studies to date. Despite the heterogeneity of ARDS, potential adverse events may also compromise safety or adherence. Cell-based therapy gradually gained the attention of researchers due to its effective control of disease initiation and progression by cell replacement, including autologous hematopoietic stem cells, mesenchymal, and related stem cells. While early-phase clinical trials suggest that the allogeneic administration of mesenchymal stem/stromal cells is safe, considerable challenges exist in moving forward to phase III efficacy studies (11). Tregs, as an immune regulatory cell, is extremely suitable for allogeneic administration; however, the mechanism underlying the use of Tregs for clinical treatment requires further exploration. In this study, we found that despite the frequency of Tregs remained unchanged, the rate of CD39^+^ Tregs was significantly downregulated following inflammation.

Increasing information indicates that distinctive Treg subtypes seem to play a different role in controlling the immune system. CD39 is an ectoenzyme that hydrolyses ATP and ADP to AMP and exhibits immunosuppressive effects. CD39 is considered to be a Treg marker because of its important regulatory function; however, whether CD39^+^ Tregs are functional in ARDS and the associated underlying mechanisms remains unknown. Utilizing CD39 knockout mice models and Treg transplantation methods, we demonstrated that allotransplanted CD39^+^ Tregs significantly relieved LPS-induced inflammation in the lung, as well as the secretion of IL-6, IL-1β, and TNF-α. Moreover, adenosine incubation rescued these effects *in vitro*. Thus, we concluded that CD39^+^ Tregs restrained LPS-induced ALI *via* converting ATP/ADP into adenosine.

Recent studies have demonstrated a strong relationship between autophagy and CD39 expression. The role for autophagy in the regulation of ATP-CD39 axis in a murine model of lung cancer has previously been defined (35), and knockout of autophagy genes has been demonstrated to increase the level of CD39 expression on tumor cells (36). Furthermore, the level of autophagy and ROS modulate CD39 upregulation in Tregs (31). Thus, we evaluated the level of cellular autophagy through confocal microscopy after transfecting Tregs with GFP-mRFP-LC3 reporter plasmids. As expected, the level of cellular autophagy was significantly increased in Tregs following LPS stimulation along with a decrease in CD39 expression. Accordingly, a knockdown of autophagic-related genes abrogated this effect on CD39 expression. These results indicated that CD39 expression was at least partially suppressed by LPS-induced autophagy in Tregs. It is important to note that we also detected a significant upregulation in the level of ERK phosphorylation in CD39^+^ Tregs. This finding is consistent with a previous study that found a positive relationship between CD39 and phosphorylated ERK in myocardial ischemia/reperfusion (37). In addition, CD39 expression was significantly downregulated after the addition of the p-ERK1/2 inhibitor, LY3214996. This leads us to speculate that the phosphorylation of ERK1/2 also induced CD39 expression. According to a bioinformatic analysis integrated with the Jaspar and GO databases, we determined that there are two potential binding sites of FOS on the CD39 promoter, which is a transcriptional factor that is activated downstream of ERK. A promoter reporter luciferase assay and ChIP assay were used to determine that the GGTAATTCATG (−685 bp to −675 bp) region on the CD39 promoter was the direct binding site of FOS. Together, this evidence reveals that except for autophagy-mediated inhibition, CD39 could also induce its self-expression *via* activation of the ERK/FOS pathway, which further enhanced the inflammation-suppressive effect of CD39^+^ Tregs on ALI.

For the first time, the findings of this study demonstrated that LPS-induced inflammation in the lung inhibited CD39 expression in Tregs *via* autophagy. This contributes to the functional impairment of Tregs in immunological surveillance. Adoptive transfer of CD39^+^ Tregs restrains LPS-induced ALI *via* catabolizing ATP/ADP to adenosine. In addition, CD39 was also observed to increase its self-transcription *via* activating the ERK/FOS pathway, which formed a feedback loop in Tregs and resisted the impairment of LPS-induced inflammation. Although further research is required, CD39^+^ Treg-based therapeutics may be an avenue for the early diagnosis, prevention, and precise treatment for ARDS.

## Data Availability Statement

The original contributions presented in the study are included in the article/**Supplementary Material**. Further inquiries can be directed to the corresponding authors.

## Ethics Statement

The studies involving human participants were reviewed and approved by Jinling Hospital Ethics Review Committee. The patients/participants provided their written informed consent to participate in this study. The animal study was reviewed and approved by Institutional Animal Care and Use Committee of Jingling Hospital.

## Author Contributions

YS and FZ conceived and designed the experiments. CC carried out most of the experiments. SZ helped with the animal modeling. XL, CL, DW, and YZ contributed to clinical sample collection. CC and JJ analyzed the data. CC, FZ, and YS interpreted the results. CC, YG, and MC prepared figures. CC drafted the manuscript. HL, TL, FZ, and YS revised the manuscript. All authors contributed to the article and approved the submitted version.

## Funding

This work was supported by the National Natural Science Foundation of China (Grant No. 81970034, 81570025, 81770082, and 81570078).

## Conflict of Interest

The authors declare that the research was conducted in the absence of any commercial or financial relationships that could be construed as a potential conflict of interest.
