# Supplementary material for: CD39+ Regulatory T Cells Attenuate Lipopolysaccharide-Induced Acute Lung Injury via Autophagy and the ERK/FOS Pathway
Source: Front Immunol. 2021 Jan 8;11:602605. doi: 10.3389/fimmu.2020.602605 (PMC7819860; doi:10.3389/fimmu.2020.602605)
Supplement: Supplementary file 1 [file Table_1.docx]

Supplementary Material

# Supplementary Figures


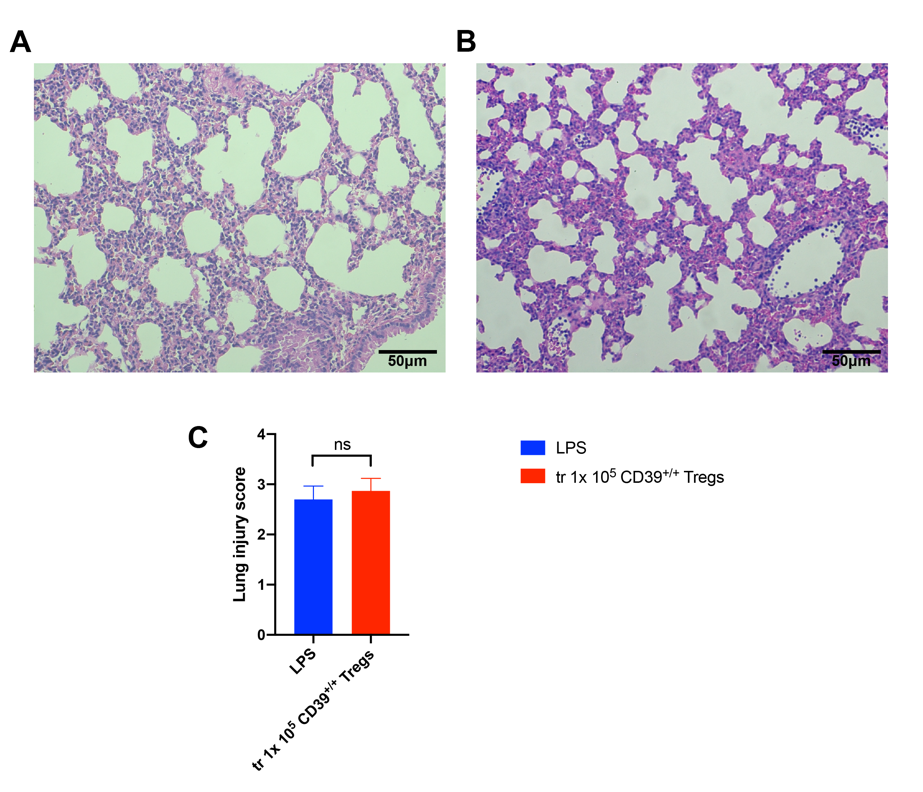


**Supplementary Figure 1.** (**A**) H&E staining of the lung tissue of LPS-WT mice (400X). (**B**) H&E staining of the lung tissue of the mice receiving 1 x 10^5^ Tregs (400X). (**C**) The lung injury score shows no difference in the two group(n=3).

**
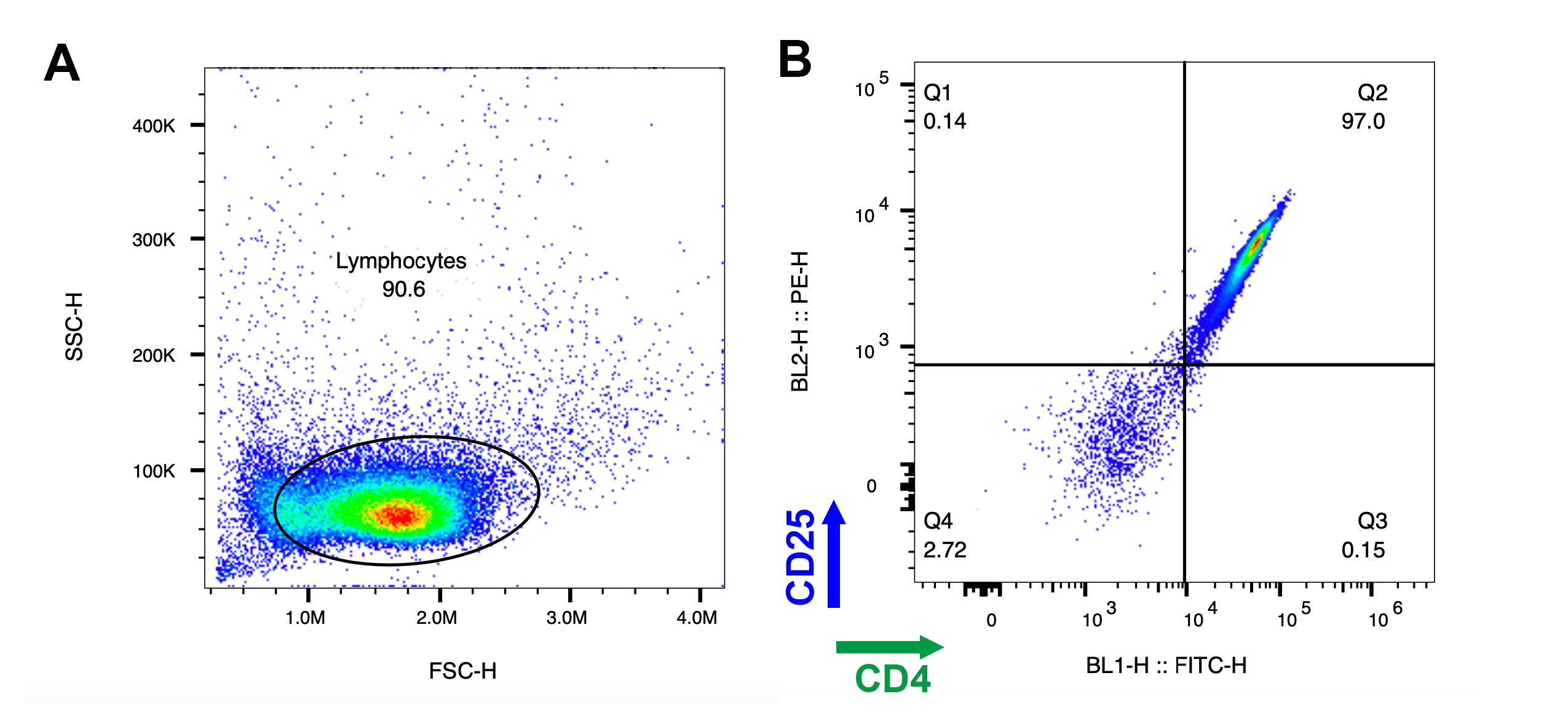
Supplementary Figure 2.** (**A-B**) Tregs were isolated by CD4^+^CD25^+^ Regulatory T-cell Isolation Kit according to the manufacturer’s recommendations, and the purity of Tregs was validated by Flow Cytometry.


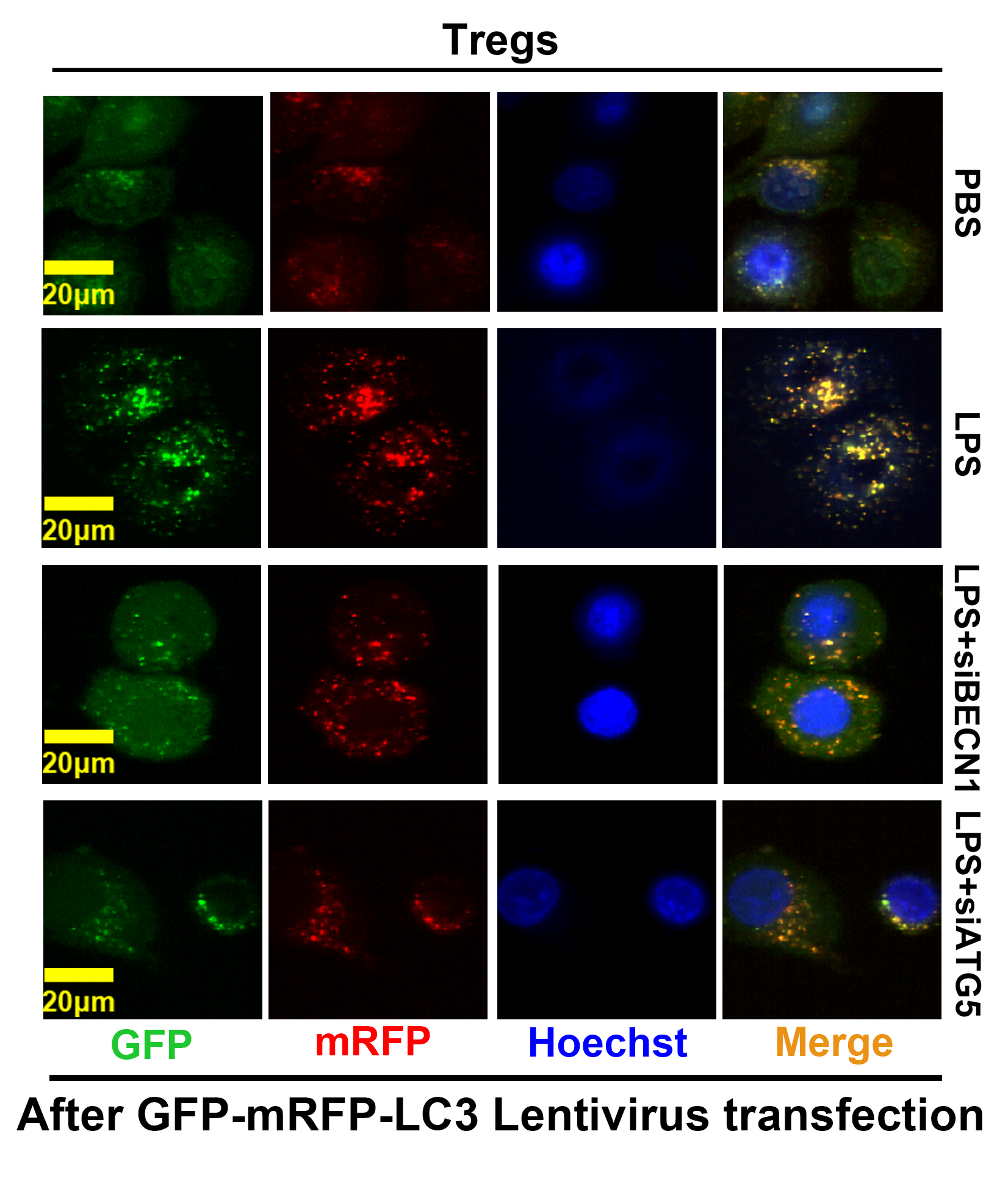


**Supplementary Figure 3.** After isolation and activation in vitro, Tregs transfected with the GFP-mRFP-LC3 lentivirus were treated with LPS or PBS in a co-culture system. Confocal microscopy was performed to determine the level of LC3 expression.


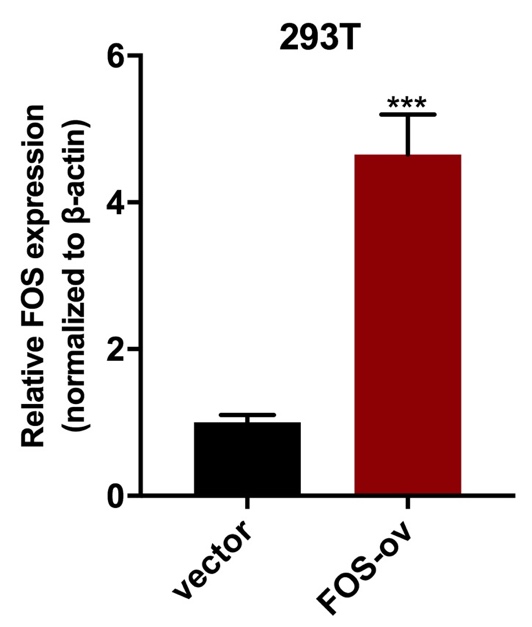


**Supplementary Figure 4.** 293T cells was transfected with FOS overexpression or control plasmids, and the expression of FOS was confirmed by RT-PCR.

| **TF** | **Source** | **Start** | | **Stop** | | **Score** | | **P-value** | | **Q-value** | | **Matched Sequence** | |
| --- | --- | --- | --- | --- | --- | --- | --- | --- | --- | --- | --- | --- | --- |
| FOS | hTFtarget | | 273 | | 288 | | 13.0746 | | 1.25E-05 | | 0.0481 | | CTACCCCTCAGCCACT |
| FOS | hTFtarget | | 1443 | | 1454 | | 11.8429 | | 5.28E-05 | | 0.21 | | CAACCAATAACG |

**Supplementary Table 1.** The binding site information of FOS on the CD39 promoter based predicted by the hTFtarget database.
